# Willingness for Medical Screening in a Dental Setting—A Pilot Questionnaire Study

**DOI:** 10.3390/ijerph20216969

**Published:** 2023-10-24

**Authors:** Asiye Özcan, Nina Nijland, Victor E. A. Gerdes, Josef J. M. Bruers, Bruno G. Loos

**Affiliations:** 1 Department of Periodontology, Academic Center for Dentistry Amsterdam (ACTA), University of Amsterdam and Vrije Universiteit Amsterdam, 1081 LA Amsterdam, The Netherlandsb.loos@acta.nl (B.G.L.); 2 Department of Internal Medicine, Amsterdam University Medical Center (AUMC), University of Amsterdam and Vrije Universiteit Amsterdam, 1105 AZ Amsterdam, The Netherlands; 3 Department of Internal Medicine, Spaarne Gasthuis, 2134 TM Hoofddorp, The Netherlands; 4 Department of Oral Public Health, Academic Centre for Dentistry Amsterdam (ACTA), University of Amsterdam and Vrije Universiteit Amsterdam, 1081 LA Amsterdam, The Netherlands; 5 Royal Dutch Dental Association (KNMT), 3528 BB Utrecht, The Netherlands

**Keywords:** noncommunicable diseases, cardiovascular diseases, diabetes mellitus, medical screening, dental clinic, health check

## Abstract

An important way to manage noncommunicable diseases (NCDs) is to focus on prevention, early detection, and reducing associated risk factors. Risk factors can be detected with simple general health checks, which can also be performed in dental clinics. The purpose of this study was to investigate participants’ willingness to participate in general health checks at the dentist, in particular the difference in opinion between medical patients and random healthy dental attendees. A total of 100 medical patients from an outpatient internal medicine clinic and 100 dental clinic attendees were included (total of 200 participants). The participants were asked for their opinion using six closed-ended questions. Overall, 91.0% of participants were receptive to information about the risk of diabetes mellitus (DM) and cardiovascular diseases (CVD). The majority (80–90%) was receptive to screening for DM and CVD risk, such as weight and height measurements, blood pressure measurement, saliva testing for CVD and to measure glucose and cholesterol via finger stick. No significant differences were found in the frequencies of the responses between the different groups based on health status, age, sex, or cultural background. This study shows that most participants are willing to undergo medical screening at the dentist for early detection and/or prevention of common NCDs.

## 1. Introduction

Noncommunicable diseases (NCDs) cause major health problems worldwide [1]. The main types of NCDs are cardiovascular diseases (CVDs), cancers, chronic respiratory diseases and diabetes mellitus (DM). The prevalence of NCDs is growing globally, and their impact on the global disease burden and healthcare economy is considerable [2]. High blood pressure, obesity and metabolic deviations, such as hypercholesterolemia, hyperglycemia and hyperlipidemia, increase the risk of NCDs [3,4]. Not only the metabolic risk factors, but also some lifestyle factors, such as tobacco use, physical inactivity, unhealthy diet and the harmful use of alcohol, increase the risk of NCDs [3,4]. Also, it is now recognized that many people with one type of NCD suffer simultaneously from another NCD, and this risk increases with age [5]. Having two or more diseases is called “multimorbidity”, and multimorbidity is on the rise in Europe [6,7].

Of the NCDs, DM is a group of metabolic diseases characterized by hyperglycemia that results from defects in insulin secretion, insulin sensitivity, or both [8,9]. There are 537 million adults living with DM around the world [10]. DM was responsible for 6.7 million deaths in 2021 [10]. Furthermore, DM caused at least USD 966 billion in health expenditure—a 316% increase over the last 15 years [10]. In 2021, almost one in two adults (20–79 years old) with diabetes was unaware of their diabetes status (44.7%) [11]. Type 2 diabetes (T2DM) is the most common type of DM [12]. T2DM goes often undiagnosed for many years because hyperglycemia develops slowly [13]. At earlier stages (prediabetes stages) the classic symptoms of DM are not severe enough for the patient to notice [8,13,14]. Nevertheless, such patients are at increased risk of developing the complications of DM [14]. These are classified into microvascular and macrovascular diseases [15]. Microvascular complications include retinopathy, neuropathy and nephropathy [16]. Macrovascular diseases concern CVDs, like coronary artery disease [17,18,19].

According to the World Health Organization (WHO), CVDs account for most deaths due to NCDs, with 17.9 million people in 2019 [20]. This represents 32% of all global deaths [20]. CVDs are a group of blood vessel disorders affecting the heart, brain and other organs. There are different types of CVDs, the most common of which are coronary heart disease, stroke and peripheral arterial disease. CVD occurs when the blood supply is interrupted or blocked due to atherosclerosis [21]. Atherosclerosis is characterized by chronic inflammation and lipid accumulation within the arteries, ultimately restricting blood flow to the artery and leading to several CVDs [22,23,24]. CVDs are a type of NCDs with many risk factors, such as elevated blood pressure, hypercholesterolemia, diabetes, obesity, a family history of CVD, age and sex [25]. The awareness and prevention with non-pharmacologic approaches could reduce these risk factors [26]. Early detection and early treatment including changes in lifestyle could prevent severe complications and thus could contribute to better disease management [25,26,27,28,29].

The evidence for associations between oral and systemic diseases has been studied by many authors [30,31,32,33,34]. As such, dental professionals are highly aware of the link between oral and systemic diseases. Many prevalent risk factors are shared between them; these risk factors include smoking, ethnicity, genetics and obesity [32,35,36]. The strongest evidence for associations between oral and systemic diseases exists between periodontitis and DM and between periodontitis and CVD [30,31,32,33,34].

Screening for risk factors for DM and CVDs could be performed by (non)invasive measurements of blood pressure, Body Mass Index (BMI), glycated hemoglobin (HbA1c) and cholesterol levels [37,38,39]. HbA1c reflects the average blood glucose levels over a duration of 8–12 weeks [40]. Saliva has also been suggested as a non-invasive screening method for common chronic diseases such as DM [41,42]. Since most patients visit their dentist more often than a medical doctor, the dental clinic can be a unique setting to perform general health checks using additional health questions, a saliva test or a finger stick blood test, blood pressure and height and weight measurements [43]. With this, patients can be informed whether they run the (increased) risk to have or to develop DM or CVD.

A recent study in Birmingham (UK) found that more than half of respondents agreed with the concept of screening for NCDs in dental settings [44]. Another study in Plymouth (UK) found a patient acceptance of 87% to the concept of medical screening in dental settings [45]. Whether these findings hold true in the setting of another country is crucial because successful execution of screening for NCDs in a dental setting requires the interest and collaboration of probable users of the service. To the best of our knowledge, firstly, there is no research in the Netherlands regarding patients’ attitude about general health checks at the dental clinic. Second, due to the healthcare system in the Netherlands, most patients visit the dentist more regularly than their general practitioner, which could result in a different outcome in our country compared to other European countries. Third, in our country there is a relatively large group of culturally non-Dutch inhabitants; this may also affect outcome results. The primary aim of the current study was to investigate the willingness of medical and dental patients to undergo a simple health check in a dental setting. The secondary aim was to investigate whether there is a difference between the opinion of medical patients in the outpatient medical setting and presumed healthy individuals in the dental clinics. We expected variation in attitudes and willingness about the health check in a dental clinic depending on the questioned invasiveness of the screening tests.

## 2. Materials and Methods

### 2.1. Study Design

The present study was carried out as cross-sectional research following the STROBE guidelines [46] between April and June 2022 by using an anonymous, written questionnaire. Prior to the study, ethical clearance was given by the Internal Review Board of the Academic Center of Dentistry Amsterdam (ACTA) with document number 2022-84448.

### 2.2. Settings and Participants

**Figure 1:**
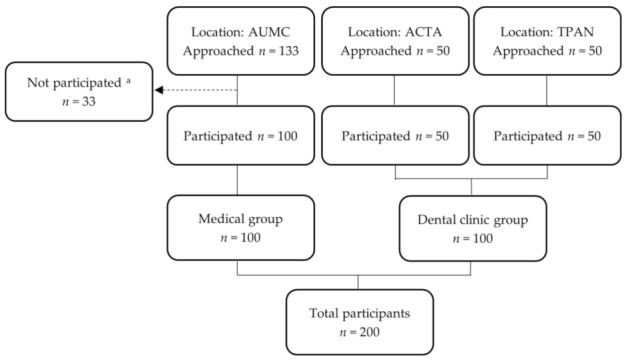
Flowchart of the study setting with the locations and number of participants. ^a^ Reasons for not participating in the study: lack of time and disinterest. Abbreviations: *n* = number; AUMC = Amsterdam University Medical Center; ACTA = Academic Center of Dentistry Amsterdam; TPAN = Tandartsenpraktijk Amsterdam-Noord.

The study was performed at three locations in Amsterdam, in two different settings, with two equal groups of 100 participants each: a group of “medical patients” and a group of healthy dental attendees, from here on called “dental clinic group” (Figure 1). Potential participants under the age of 18 years and non-Dutch speakers were not eligible. Participants of the “medical group” were approached in the waiting room of the outpatient clinic of the Department of Internal Medicine in the AUMC (Amsterdam University Medical Center, location AMC). These patients represented the group already having one or more diseases. Upon arrival before their consultation at the medical specialist, in consecutive order, patients were asked if they were willing to participate in answering research questions. If so, informed consent was signed. Thereafter, demographic data (age, sex and cultural background (Dutch, non-Dutch)) were collected through four questions, and patients were asked to complete the questionnaire. The prevalence of DM, CVD, and/or other diseases was retrieved from the medical records.

Half of the participants of the “dental clinic group” were approached in the dental clinics of the ACTA and the other half in a general dental clinic in Amsterdam (Tandartsenpraktijk Amsterdam Noord (TPAN)), at both places again in consecutive order. The general health status was assessed by using the American Society of Anesthesiologists (ASA) classification [47]. Only individuals classified as ASA I (normal healthy, non-smoking) were eligible for participation in the “dental clinic group” to ensure that this group was without any history of one or more illnesses or NCDs. When the dental attendees met the inclusion criteria and were willing to participate, informed consent was signed. Thereafter, participants were asked to fill in the questionnaire.

### 2.3. Questionnaire

The first questions of the questionnaire were devised to collect the demographic data. The questions “Are you born in the Netherlands?” and “Are both of your parents born in the Netherlands?” were used to categorize the cultural background. If both questions were answered with ‘yes’, the participants were categorized as ‘culturally Dutch’; otherwise, if one or both questions were answered with ‘no’, the participants were categorized as culturally non-Dutch’. To investigate participants’ opinion about a general health check at the dentist, six closed-ended questions with ‘yes’ or ‘no’ as answer possibilities were formulated. The following questions were asked:

- Are you open to having the dentist inform you whether you are at risk of developing diseases, such as diabetes and cardiovascular diseases?
- Are you open to having your dentist take a saliva test to determine the risk of cardiovascular disease?
- Are you open to having the dentist measure your blood pressure?
- Are you open to having the dentist measure your weight and height (BMI)?
- Are you open to having the dentist measure your blood glucose level using a finger prick?
- Are you open to having the dentist measure your cholesterol using a finger prick?

The paper questionnaires were digitized in Qualtrics software (Qualtrics, Provo, UT, USA) and thereafter stored in folders in a closed closet at the Department of Periodontology at ACTA (Appendix A).

### 2.4. Statistical Analysis

No power calculation was performed for the current research question. This decision stemmed from our lack of a priori knowledge concerning the willingness of the dental attendees to participate in this survey on the topic of medical screening in a dental setting. Therefore, the results are considered preliminary and explorative.

Statistical analyses were performed in IBM SPSS Statistics version 28.0 (IBM, New York, NY, USA). Differences in background data and responses to the willingness for medical screening in a dental setting and questions between the two groups of patients were analyzed with a Chi-square test or an independent sample *t*-test, depending on the level of measurement. In addition, it was examined for both groups of patients together whether the willingness for medical screening in a dental setting showed a relationship with age (based on the median), with gender (male and female) and with cultural background (cultural Dutch or cultural non-Dutch) of the patients. These analyses were also performed with a Chi-square test. For all analyses, a *p*-value of < 0.05 was considered statistically significant.

## 3. Results

### 3.1. Background Data

In total, 200 persons participated and completed the questionnaires, *n* = 100 in the medical group and *n* = 100 in the dental clinic group (Figure 1). The reasons for not participating in the study were: a lack of time or not willing to participate.

The background data for all participants and for the two groups are presented in Table 1. As can be seen, the average age in the medical group is higher than in the dental clinic group (55.9 ± 16.2 vs. 39.6 ± 15.6, *p* < 0.001). The proportion of men in this group was also higher (57% vs. 47%, *p* = 0.157), but this difference was not statistically significant. However, this does apply to the difference in cultural background, where it appears that in the medical group a larger proportion had a culturally Dutch background (71% vs. 39%; *p* < 0.001). In the medical group, 20 participants (20.0%) had T2DM, 21 participants (21.0%) had CVD, 6 participants (6.0%) had both T2DM and CVD and 65 participants (65.0%) had other diseases.

### 3.2. Primary Results

The results of the questions about a general health check at the dentist are shown in Table 2. In total, 91.0% of participants were open to receiving information from their dentist about the risk of developing DM and CVD. In addition, 90.5% were willing to undergo a saliva test to detect the risk of developing CVD. Furthermore, 89.0% were open to blood pressure measurement and 80.5% to weight and height measurements. Finally, 80.0% of all participants indicated that they were open to a finger prick to measure blood glucose and 80.0% to measure cholesterol levels. There were no statistically significant differences between participants in the medical group and the dental clinic group.

### 3.3. Secondary Results

The frequencies of the responses to the questionnaire were analyzed between the subgroups of age, sex and cultural background (see Supplementary Tables S1–S3 in Supplementary Materials). The willingness to undergo health checks at the dental clinic did not differ by age. An exception was the willingness to have blood pressure measured at the dental clinic, which was higher in patients >50 years than in patients younger than 50 years (94.1% vs. 83.8%; *p* = 0.021). There were also no statistically significant differences regarding sex and cultural background. Exploratory analysis between the frequencies of the responses to the questionnaire between the different locations of the dental clinic group showed similar trends, and no significant differences were found (see Supplementary Table S4 in Supplementary Materials).

### 3.4. Additional Comments

Unsolicited additional comments were also provided by some participants. In total, 18 participants (9.0%) did not wish to be informed about risk of DM and CVD development by their dentist. The comment most given was that they did not want to know if they were at risk. Another given reason was that the dentist is not educated in this.

In total, 19 participants (9.5%) were not open to a saliva test. One reason that was mentioned for not being open to a saliva test was the perceived possibility of DNA collection through saliva testing. The most common comment given by participants who were not open to blood pressure measurement, to the height and weight measurements, and/or to testing blood glucose and cholesterol level at the dental clinic was that dentists should concentrate on their own work, and that this kind of measurements should be done at the family doctor’s office. Some participants of the medical group did not desire testing at the dental clinic, as they already conducted these tests at home or in the hospital. In general, the expected costs were mentioned as a reason to refrain from testing at the dental clinic.

## 4. Discussion

### 4.1. Key Results

The primary aim of this pilot study was to obtain preliminary evidence about attitudes towards a general health check and additional blood or saliva testing within a dental setting. In general, the responses to the questions were mostly positive. The secondary aim was to investigate whether there was a difference in willingness for medical screening between medical patients under treatment at the outpatient internal medicine department and dental clinic attendees. There were no significant differences between these groups. More than three quarters of the participants in both groups were willing to undergo medical screenings at the dental clinic. Further analyses of the subgroups based on age, sex and cultural background did not reveal relevant differences either.

### 4.2. Interpretation

The results of the current study were highly comparable to those from a study performed in Plymouth (UK) [45]. The study investigated dental patients’ attitudes and acceptance towards medical screening, including diabetes, within dental settings. They used a questionnaire with five closed-ended questions. The vast majority, 87% of the participants, reported that it was important that dentists screen patients for medical conditions and 79% were very willing to let a dental team member carry out screening. This was further supported by a study of Greenberg et al. (2012) performed in the United States of America (USA) [48]. Most of the surveyed patients felt that it was important that dentists could screen for conditions for which patients are unaware of and to monitor medical conditions that patients are suffering from (87% and 91%, respectively). Furthermore, 84–88% of the respondents were willing to undergo blood pressure measurements and weight and height measurements, as well as to provide oral fluids and finger stick blood sampling (84% and 70%, respectively). Our findings are also closely aligned with the results of the Yonel et al. study performed in Birmingham, UK; they confirmed the public support for medical screening in dental settings [44].

The systematic review of Yonel et al. (2020) reported some barriers to implement screening for non-diabetic hyperglycaemia and undiagnosed T2DM in adults within the dental practice [49]. The studies in the review cited time, cost and lack of insurance cover as barriers [49]. These findings are consistent with the opinion of the participants in our study, although we did not ask this systematically. The American study of Greenberg et al. (2012) showed that 64% of dental patients were open to pay USD 10–20, and 50% was willing to pay USD 21–30 for chairside medical screenings at the dental clinic [48].

Screening for risk factors of DM and CVD during dental visits has the ability to raise patients’ awareness of their risk for such NCDs [50]. NCDs, especially DM and CVD are generally discovered late, usually because complications have developed [27,51,52]. The early detection of the risk factors, early diagnosis and treatment are important to start management with counseling and pharmacotherapy in time. This has additional positive effects in reducing the associated public health cost [1,27].

It is also interesting to consider the viewpoints of the dentists, governments, and dental organizations about the concept of general health checks at the dental clinic. The study of Greenberg et al. (2010) and Curran et al. (2014) suggested that most dentists are willing to conduct screening for medical conditions or to address medical conditions [53,54]. The study of Friman et al. (2015) showed that the authorities and organizations in Sweden generally had a positive view of general health checks and medical screenings at the dental clinic [55]. Nevertheless, they were uncertain about the concept. Further investigations are needed to assess the feasibility, (cost-)effectiveness and operational challenges in the Netherlands. Unpublished results from a 2016 survey study among dentists in the Netherlands about their attitude towards medical screening of their patients showed divided opinions: a third responded positive about the concept, a third neutral and another third negative.

### 4.3. Strengths and Limitations

This study has some strengths. First, the questionnaire that was used in this study was face validated. It was designed by an expert epidemiologist to measure the relevance and appropriateness of the issue. Second, to increase the heterogeneity of the dental clinic group, we purposely decided to approach these at two different locations, one academic and one private. Thirdly, in order to have enough information on persons with or without NCD, two different groups were created. The medical group included patients who have at least one NCD; on the other hand, the group of dental attendees included only participants who do not have a history of any known NCDs. The group of dental attendees may benefit more from general health checks at the dentist. Yet another strength of the current study is the inclusion of sufficient numbers of culturally non-Dutch participants; they represent a major group in the greater Amsterdam area seeking medical and dental care.

The current study also has limitations. The medical group and the dental clinic group were not equal in terms of age and cultural background. The participants in the medical setting were older and this setting had more participants with a Dutch cultural background. Second, we may have encountered volunteer bias (also called self-selection bias) in this study; due to the explorative nature of the study, the current convenience sample was constructed. Further, the questionnaire determined whether the participants were open to the measurements taken specifically by their dentist and not by other dental professionals, such as the dental hygienists. Dental hygienists have an important role as well in general dental healthcare. The measurements could also be carried out by them, but they were not mentioned in the questionnaire. Another limitation can be considered that we did not include additional questions from previous studies [44,45] which included: “Are you registered with a dentist?”, “How regularly do you visit the dentist?”, “Are you open to a health check of your lungs, your kidneys and to check the Vitamin D levels?”. These additional questions could make comparisons with previous studies on the same topic more complete, although our results are comparable.

### 4.4. Generalizability

The generalizability of the current results may be somewhat limited. The study is a pilot study in the Netherlands, performed in the greater Amsterdam area. However, it is encouraging that our results are very similar to the results in the UK and the USA. Therefore, we suggest that the current results may have value for the whole of the Netherlands and perhaps for Western Europe. But large-scale studies like the current one are needed that include multiple geographical locations in the Netherlands, as well as including similar questions as in previous publications to confirm the suggestions. Also, these should include the dental profession to inquire about their attitudes and opinions.

## 5. Conclusions

In conclusion, the results of this pilot study show high patient willingness and interest in medical screenings in a dental setting. The majority of participants showed a positive opinion to general health checks in a dental setting. Patients’ approval and acceptance is an important step for successful medical screening in dental settings to manage noncommunicable diseases with a focus on prevention, early detection and on reducing the associated risk factors.

## Figures and Tables

**Table 1:** Background characteristics of the study population.

Characteristics	Total Study Population (*n* = 200)	Medical Group (*n* = 100)	Dental Clinic Group (*n* = 100)	*p*-Value *
Age (years)	47.7 ± 17.8	55.9 ± 16.2	39.6 ± 15.6	<0.001
Sex				
Male	104 (52.0)	57 (57.0)	47 (47.0)	0.157
Female	96 (48.0)	43 (43.0)	53 (53.0)	
Cultural background				
Dutch	110 (55.0)	71 (71.0)	39 (39.0)	<0.001
Non-Dutch	90 (45.0)	29 (29.0)	61 (61.0)	
T2DM ^a^				
Yes	20 (10.0)	20 (20.0)	0 (0.0)	N.T.
No	180 (90.0)	80 (80.0)	0 (0.0)	
CVD ^a^				
Yes	21 (10.5)	21 (21.0)	0 (0.0)	N.T.
No	179 (89.5)	79 (79.0)	0 (0.0)	
Other diseases	65 (32.5)	65 (65.0)	0 (0.0)	N.T.

Data are presented as either mean ± SD or *n* (%). * Independent sample *t*-test or chi-square test was used to analyze differences between the medical group and dental clinic group. ^a^ Six participants had both T2DM and CVD. Abbreviations: *n* = number; T2DM = Type 2 Diabetes Mellitus; CVD = cardiovascular disease. N.T. = difference not tested.

**Table 2:** Frequencies of responses to the questionnaire for the total population and for the medical group and dental clinic group.

Questions	Total Study Population (*n* = 200)	Medical Group (*n* = 100)	Dental Clinic Group (*n* = 100)	*p*-Value *
Q1. Are you open to having the dentist inform you whether you are at risk of developing diseases, such as diabetes and cardiovascular diseases?				
Yes No	182 (91.0) 18 (9.0)	91 (91.0) 9 (9.0)	91 (91.0) 9 (9.0)	1.000
Q2. Are you open to having your dentist take a saliva test to determine the risk of cardiovascular disease?				
Yes No	181 (90.5) 19 (9.5)	94 (94.0) 6 (6.0)	87 (87.0) 13 (13.0)	0.091
Q3. Are you open to having the dentist measure your blood pressure?				
Yes No	178 (89.0) 22 (11.0)	87 (87.0) 13 (13.0)	91 (91.0) 9 (9.0)	0.366
Q4. Are you open to having the dentist measure your weight and height (BMI)?				
Yes No	161 (80.5) 39 (19.5)	82 (82.0) 18 (18.0)	79 (79.0) 21 (21.0)	0.592
Q5. Are you open to having the dentist measure your blood glucose level using a finger prick?				
Yes No	160 (80.0) 40 (20.0)	82 (82.0) 18 (18.0)	78 (78.0) 22 (22.0)	0.480
Q6. Are you open to having the dentist measure your cholesterol using a finger prick?				
Yes No	160 (80.0) 40 (20.0)	84 (84.0) 16 (16.0)	76 (76.0) 24 (24.0)	0.157

Data are presented as *n* (%); *n* = number; * Chi-square tests were used to analyze differences between the medical group and dental clinic group.

## Data Availability

The data presented in this study are available on request from the corresponding author.

## Supplementary Materials

The following supporting information can be downloaded at: https://www.mdpi.com/article/10.3390/ijerph20216969/s1, Supplementary Table S1: Frequencies of responses to the questionnaire between the age subgroups; Table S2: Frequencies of responses to the questionnaire between males and females; Table S3: Frequencies of responses to the questionnaire between culturally Dutch and culturally non-Dutch participants; Table S4: Frequencies of responses to the questionnaire between the different locations of the dental clinic group.

## Appendix A. Questionnaire with Their Dutch Translations (Italic)

Most people visit the dentist every year for a periodic examination. Dentists can see signs of diabetes and cardiovascular disease in the mouth. In addition, it is conceivable that a few simple checks can be carried out in the dental clinic to recognize signs of these and other diseases. We would like to ask you some questions about this.

*De meeste mensen gaan jaarlijks naar de tandarts voor een periodiek mondonderzoek. Tandartsen kunnen in de mond signalen zien van beginnende suikerziekte en hart- en vaatziekten. Daarnaast is denkbaar dat in de tandartspraktijk enkele eenvoudige controles kunnen worden uitgevoerd om signalen van deze en andere ziekten te herkennen. Hierover willen we u graag enkele vragen stellen.*

1. Age/*Leeftijd*:
2. Sex/Geslacht:  o Female/*Vrouw* o Male/Man  o Other/*Anderszins*  o Rather not say/*Wens dit niet aan te geven*
3. Are you born in the Netherlands?/*Bent u in Nederland geboren?*
  ○ Yes/*Ja*
  ○ No/*Nee*
4. Were both your parents born in the Netherlands?/*Zijn uw beide ouders in Nederland geboren?*
  ○ Yes/*Ja*
  ○ No/*Nee*
5. Are you open to having the dentist inform you whether you are at risk of developing diseases, such as diabetes and cardiovascular diseases?/*Staat u er voor open dat de tandarts u informeert als u risico loopt op het krijgen van mogelijke ziektes zoals diabetes en hart- en vaatziekten?*
  ○ Yes/*Ja*
  ○ No/*Nee*
6. Are you open to having your dentist take a saliva test to determine the risk of cardiovascular disease?/*Staat u er voor open dat de tandarts bij u een speekseltest afneemt om het risico op hart- en vaatziekten vast te stellen?*
  ○ Yes/*Ja*
  ○ No/*Nee*
7. Are you open to having the dentist measure your blood pressure?/*Staat u er voor open dat de tandarts uw bloeddruk meet?*
  ○ Yes/*Ja*
  ○ No/*Nee*
8. Are you open to having the dentist measure your weight and height (BMI)?/*Staat u er voor open dat de tandarts uw gewicht en lengte (BMI) meet?*
  ○ Yes/*Ja*
  ○ No/*Nee*
9. Are you open to having the dentist measure your blood glucose level using a finger prick?/*Staat u er voor open dat de tandarts met behulp van een vingerprik uw bloedsuikerwaarde meet?*
  ○ Yes/*Ja*
  ○ No/*Nee*
10. Are you open to having the dentist measure your cholesterol using a finger prick?/*Staat u er voor open dat de tandarts met behulp van een vingerprik uw cholesterol meet?*
  ○ Yes/*Ja*
  ○ No/*Nee*
