# Supplementary material for: Willingness for Medical Screening in a Dental Setting—A Pilot Questionnaire Study
Source: Int J Environ Res Public Health. 2023 Oct 24;20(21):6969. doi: 10.3390/ijerph20216969 (PMC10650185; doi:10.3390/ijerph20216969)
Supplement: Supplementary file 1 [file ijerph-20-06969-s001.zip › ijerph-2552908-supplementary.pdf]

International Journal of
*Environmental Research and Public Health*

## Supplementary tables

**Table S1.** Frequencies of responses to the questionnaire between the age subgroups.

| Questions | <50 Years of Age (*n* = 101) | ≥50 Years of Age (*n* = 99) | *p*-Value * |
|---|---|---|---|
| Q1. Are you open to having the dentist inform you whether you are at risk of developing diseases, such as diabetes and cardiovascular diseases? | | | |
| Yes | 93 (92.1) | 89 (89.9) | 0.590 |
| No | 8 (7.9) | 10 (10.1) | |
| Q2. Are you open to having your dentist take a saliva test to determine the risk of cardiovascular disease? | | | |
| Yes | 90 (89.1) | 91 (91.9) | 0.498 |
| No | 11 (10.9) | 8 (8.1) | |
| Q3. Are you open to having the dentist measure your blood pressure? | | | |
| Yes | 95 (94.1) | 83 (83.8) | 0.021 |
| No | 6 (5.9) | 16 (16.2) | |
| Q4. Are you open to having the dentist measure your weight and height (BMI)? | | | |
| Yes | 80 (79.2) | 81 (81.8) | 0.641 |
| No | 21 (20.8) | 18 (18.2) | |
| Q5. Are you open to having the dentist measure your blood glucose level using a finger prick? | | | |
| Yes | 81 (80.2) | 79 (79.8) | 0.944 |
| No | 20 (19.8) | 20 (20.2) | |
| Q6. Are you open to having the dentist measure your cholesterol using a finger prick? | | | |
| Yes | 79 (78.2) | 81 (81.8) | 0.524 |
| No | 22 (21.8) | 18 (18.2) | |

Data are presented as *n* (%); *n* = number; * Chi-square tests were used to analyze differences between the age subgroups.

**Table S2.** Frequencies of responses to the questionnaire between males and females.

| Questions | Male (*n* = 104) | Female (*n* = 96) | *p*-Value * |
|---|---|---|---|
| Q1. Are you open to having the dentist inform you whether you are at risk of developing diseases, such as diabetes and cardiovascular diseases? | | | |
| Yes | 97 (93.3) | 85 (88.5) | 0.243 |
| No | 7 (6.7) | 11 (11.5) | |
| Q2. Are you open to having your dentist take a saliva test to determine the risk of cardiovascular disease? | | | |
| Yes | 95 (91.3) | 86 (89.6) | 0.671 |
| No | 9 (8.7) | 10 (10.4) | |
| Q3. Are you open to having the dentist measure your blood pressure? | | | |
| Yes | 92 (88.5) | 86 (89.6) | 0.800 |
| No | 12 (11.5) | 10 (10.4) | |
| Q4. Are you open to having the dentist measure your weight and height (BMI)? | | | |
| Yes | 85 (81.7) | 76 (79.2) | 0.647 |
| No | 19 (18.3) | 20 (20.8) | |
| Q5. Are you open to having the dentist measure your blood glucose level using a finger prick? | | | |
| Yes | 81 (77.9) | 79 (82.3) | 0.436 |
| No | 23 (22.1) | 17 (17.7) | |
| Q6. Are you open to having the dentist measure your cholesterol using a finger prick? | | | |
| Yes | 82 (78.8) | 78 (81.3) | 0.671 |
| No | 22 (21.2) | 18 (18.7) | |

Data are presented as *n* (%); *n* = number; * Chi-square tests were used to analyze differences between males and females.

**Table S3.** Frequencies of responses to the questionnaire between culturally Dutch and culturally non-Dutch participants.

| Questions | Culturally Dutch *(n = 110)* | Culturally Non-Dutch *(n = 90)* | *p*-Value * |
|---|---|---|---|
| Q1. Are you open to having the dentist inform you whether you are at risk of developing diseases, such as diabetes and cardiovascular diseases? | | | |
| Yes | 101 (91.8) | 81 (90.0) | 0.655 |
| No | 9 (8.2) | 9 (10.0) | |
| Q2. Are you open to having your dentist take a saliva test to determine the risk of cardiovascular disease? | | | |
| Yes | 102 (92.7) | 79 (87.8) | 0.235 |
| No | 8 (7.3) | 11 (12.2) | |
| Q3. Are you open to having the dentist measure your blood pressure? | | | |
| Yes | 96 (87.3) | 82 (91.1) | 0.388 |
| No | 14 (12.7) | 8 (8.9) | |
| Q4. Are you open to having the dentist measure your weight and height (BMI)? | | | |
| Yes | 88 (80.0) | 73 (81.1) | 0.844 |
| No | 22 (20.0) | 17 (18.9) | |
| Q5. Are you open to having the dentist measure your blood glucose level using a finger prick? | | | |
| Yes | 87 (79.1) | 73 (81.1) | 0.722 |
| No | 23 (20.9) | 17 (18.9) | |
| Q6. Are you open to having the dentist measure your cholesterol using a finger prick? | | | |
| Yes | 87 (79.1) | 73 (81.1) | 0.722 |
| No | 23 (20.9) | 17 (18.9) | |

Data are presented as *n* (%); *n* = number; * Chi-square tests were used to analyze differences between 'culturally Dutch' and 'culturally non-Dutch' participants.

**Table S4.** Frequencies of responses to the questionnaire between the different locations of the dental clinic group.

| Questions | Location ACTA *(n = 50)* | Location TPAN *(n = 50)* | *p*-Value * |
|---|---|---|---|
| Q1. Are you open to having the dentist inform you whether you are at risk of developing diseases, such as diabetes and cardiovascular diseases? | | | |
| Yes | 45 (90.0) | 46 (92.0) | 0.727 |
| No | 5 (10.0) | 4 (8.0) | |
| Q2. Are you open to having your dentist take a saliva test to determine the risk of cardiovascular disease? | | | |
| Yes | 45 (90.0) | 42 (84.0) | 0.372 |
| No | 5 (10.0) | 8 (16.0) | |
| Q3. Are you open to having the dentist measure your blood pressure? | | | |
| Yes | 45 (90.0) | 46 (92.0) | 0.727 |
| No | 5 (10.0) | 4 (8.0) | |
| Q4. Are you open to having the dentist measure your weight and height (BMI)? | | | |
| Yes | 40 (80.0) | 39 (78.0) | 0.806 |
| No | 10 (20.0) | 11 (22.0) | |
| Q5. Are you open to having the dentist measure your blood glucose level using a finger prick? | | | |
| Yes | 38 (76.0) | 40 (80.0) | 0.629 |
| No | 12 (24.0) | 10 (20.0) | |
| Q6. Are you open to having the dentist measure your cholesterol using a finger prick? | | | |
| Yes | 36 (72.0) | 40 (80.0) | 0.349 |
| No | 14 (28.0) | 10 (20.0) | |

Data are presented as *n* (%); * Chi-square tests were used to analyze differences between the age subgroups. Abbreviations: *n* = number; ACTA = Academic Center of Dentistry Amsterdam; TPAN = Tandartsenpraktijk Amsterdam-Noord.
